# Potential roles of claudin-3 and claudin-4 in ovarian cancer management

**DOI:** 10.1186/s43046-022-00125-4

**Published:** 2022-06-06

**Authors:** Leshanth Uthayanan, Mona El-Bahrawy

**Affiliations:** 1 Department of Metabolism, Digestion and Reproduction, Faculty of Medicine, Hammersmith Hospital, Imperial College London, London, W12 0NN UK; 2 Department of Pathology, Alexandria Faculty of Medicine, Alexandria, Egypt

**Keywords:** Claudins, Ovarian, Cancer, Biomarker, Therapy, *Clostridium perfringens*, Enterotoxin

## Abstract

**Background:**

Ovarian cancer has the highest mortality amongst all gynaecological malignancies, with around two-thirds of patients diagnosed with advanced disease due to late presentation. Furthermore, around 90% of patients develop recurrence and eventually become chemoresistant. Therefore, there is a high demand to identify biomarkers specific to this disease for screening for early detection, as well as new therapeutic targets. Tight junctions (TJs) regulate paracellular permeability and are vital in establishing epithelial cell polarity. One hallmark of tumorigenesis is the loss of TJs, with loss of cell-to-cell adhesion. Claudins are integral TJ membrane proteins, which have been found to play a critical role in maintaining the TJ’s barrier function. Furthermore, claudin-3 (CLDN3) and claudin-4 (CLDN4) are overexpressed in ovarian cancer. This article aims to explore the biological role of CLDN3 and CLDN4 and their potential in different aspects of the management of ovarian cancer.

**Main body:**

CLDN3 and CLDN4 have been shown to be effective markers for the early detection of ovarian cancer. Whilst there is difficulty in screening for both claudins in serum, their assessment by gene expression analysis and immunohistochemical methods shows promising potential as diagnostic and prognostic biomarkers for ovarian cancer. The localisation and overexpression of claudins, such as CLDN3, have been shown to correlate with poorer survival outcomes. The added value of combining claudins with other markers such as CA125 for diagnosis has also been highlighted. Therapeutically, CLDN3 and more so CLDN4 have been shown to be effective targets of *Clostridium perfringens* enterotoxin (CPE). Interestingly, CPE has also been shown to resensitise chemoresistant tumours to therapy.

**Conclusions:**

This review presents the diagnostic and prognostic potential of CLDN3 and CLDN4 and their emerging role as therapeutic targets in ovarian cancer. Clinical trials are required to validate the promising results of the in vitro and in vivo studies for CLDN3 and CLDN4, possibly adding onto current ovarian cancer management.

## Background

Ovarian cancer remains to have the highest mortality rate amongst gynaecological malignancies [1]. Ovarian cancers are predominantly 90% of epithelial subtype and include mainly serous, endometrioid, clear-cell, and mucinous carcinomas [2]. Around two-thirds of patients has advanced disease when diagnosed, due to the insidious disease onset and lack of reliable screening tests. Whilst many patients with disseminated tumours can respond to initial standard combinations of surgical and cytotoxic therapy, around 90% unfortunately develop recurrence, eventually succumbing to chemoresistant ovarian cancer [3]. Therefore, there is a need for developing biomarkers that will allow clinicians to diagnose the disease at an early stage and prior to development of distant metastasis.

Current biomarkers include carcinoembryonic antigen (CEA), cancer antigen 125 (CA125), carbohydrate antigen 19-9 (CA19-9), and human epididymis protein 4 (HE4), with the most clinically used method for detection of ovarian cancer being transvaginal ultrasonography and serum CA125. However, biomarkers such as CA125 have a relatively low specificity and low sensitivity (50–90%) [4], meaning that there is a real demand to identify and develop tumour markers for early detection with a much higher sensitivity. Such biomarkers will allow for an effective screening procedure, which may reduce mortality by 10–30% [5]. Furthermore, the need to develop targeted therapy is warranted by the chemoresistance and recurrence properties of this disease.

One hallmark of malignant transformation is the loss of epithelial tight junctions (TJs) [6]. TJs play essential roles in establishing permeability barriers in the most apical regions of the intercellular junctional complexes. They are involved with paracellular transport, cellular polarity maintenance, and recruitment of signalling proteins. TJs consequently play a role in cellular proliferation, differentiation, and regulation [6, 7]. Therefore, any deviation from the normal functioning of TJs and their permeability properties can lead to several pathological conditions, including cancer [6].

**Fig. 1:**
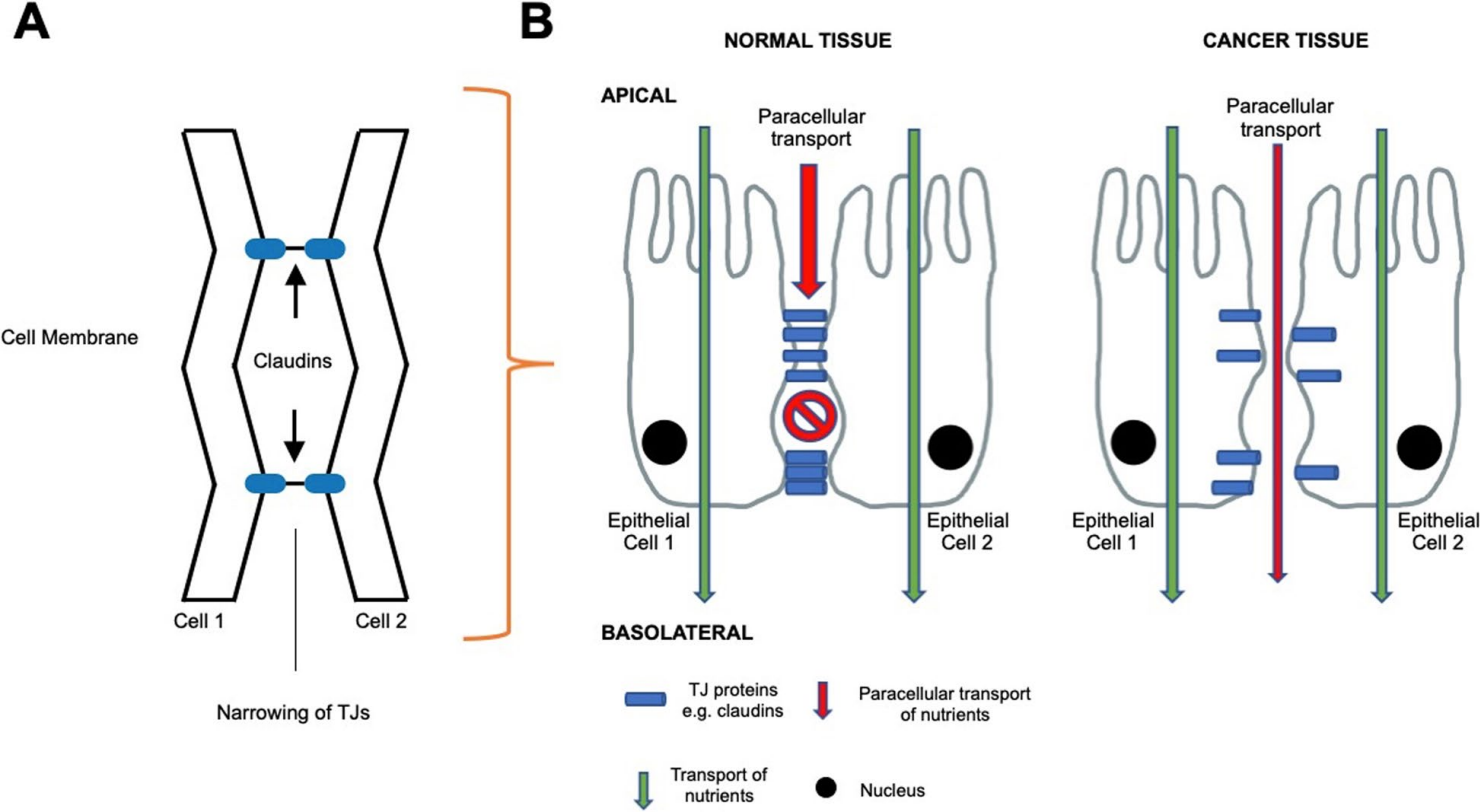
**A** Schematic view of the claudins in the TJ. The claudins are located at the membranes, narrowing the TJs, thereby increasing barrier function. **B** Epithelial cell-to-cell contact, where claudins are located at the membranes. In non-tumorigenic tissues, the TJ is intact between the cells, meaning there is controlled paracellular transport of nutrients and growth factors. When there is a loss of TJ activity, such as that seen in tumorigenesis, the barrier weakens, allowing for more liberal paracellular transport of nutrients and growth factors [6, 7, 11]

**Fig. 2:**
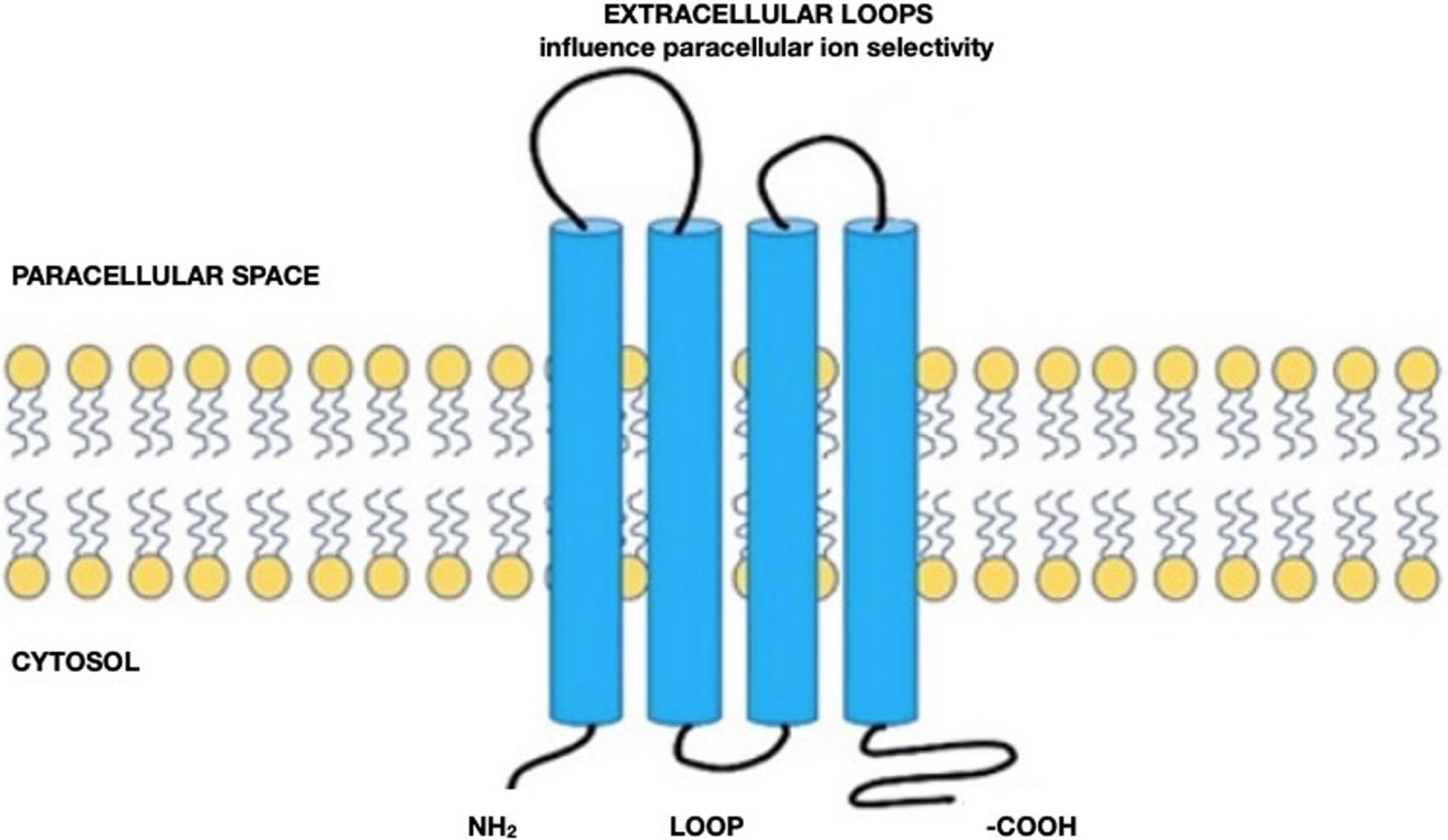
Claudin structure. Claudins consist of 4 transmembrane domains. The extracellular loops are thought to control the paracellular ion selectivity. The C-terminal has been shown to be important for signal transduction [11]

In the search for biomarkers and therapeutic targets for ovarian cancer, a serial analysis gene expression showed that claudin-3 (CLDN3) and claudin-4 (CLDN4), both significant protein components of the TJ, were amongst the most highly upregulated genes [8]. High expression of these proteins was also reported in most primary ovarian tumours in other studies [9, 10]. Claudins are major integral transmembrane proteins in the TJ, which exhibit distinct tissue- and developmental-specific distribution patterns [7] (Fig. 1). Both N- and C-terminals of claudins are located within the cytoplasm, with an extracellular loop that is thought to affect the paracellular ion selectivity [11] (Fig. 2).

The activity of CLDN3 and CLDN4 in the TJ regulation has also been associated with ovarian cancer’s response to therapy. An immunohistochemical study performed by Yoshida et al. [12] on tumours from 43 postoperative chemotherapy receiving ovarian cancer patients showed that CLDN4 was significantly elevated in the chemoresistant group. This suggests that CLDN4 may contribute to the strengthening of the TJ’s barrier function and consequently reduce cisplatin cellular accumulation. Therefore, a reduced barrier function, in response to reduced claudin activity at the TJs, may facilitate cell entrance to chemotherapeutic agents. This is supported by an increased accumulation of fluorescent-labelled cisplatin in the cells following CLDN4 inhibition by CLDN4 siRNA transfection into the human ovarian cancer cell lines OVCAR-3 and CaOV-3 [12]. Similarly, Santin et al. [13] also found significantly higher CLDN3 and CLDN4 expression in established human ovarian tumours through quantitative reverse transcription (qRT)-PCR analysis. On the other hand, in another study using human ovarian carcinoma cell line 2008, knockdown of CLDN3 and CLDN4 leads to increased resistance to cisplatin both in in vitro and in vivo xenograft models [14]. This coincided with significantly reduced endogenous copper influx transporter mRNA levels, necessary for cisplatin uptake. The differences in results may be explained by the different cell lines and methodologies used. In another in vitro study using the ovarian cancer cells lines OVCAR-4 and OVCAR-8, CLDN4 overexpression was associated with reduced paclitaxel apoptotic response [15]. Whilst previous studies have reported on CLDN4 upregulation and its association with chemotherapeutic response, they have also reported on CLDN3 to have less of an effect [14]. Moreover, another study, through immunohistochemical assessment, found no correlation between CLDN4 expression and other clinicopathological features such as chemosensitivity [16]. An interesting phenomenon observed by Santin et al. [13] is that prolonged in vitro cultures of established ovarian cancer cell lines such as OVCAR-3 and CaOV-3 showed significant downregulation of claudins when compared with primary ovarian tumours. The study also reported that advanced in vitro passages of primary ovarian serous papillary carcinoma (OSPC) had consistent downregulation of CLDN3 and CLDN4 when measured by qRT-PCR [13]. This suggests that established cancer cell lines may be suboptimal models when evaluating claudin expression in ovarian cancer. Therefore, more studies on short-term cell lines to preserve their true genetic nature [17] and in vivo studies are required to clear the controversy between the expression patterns of CLDN3 and CLDN4 and chemotherapy efficacy.

Considering the upregulation of CLDN3 and CLDN4 in ovarian cancers, their crucial role in chemotherapy response and TJ formation, and TJ’s important role in tumorigenesis, this review aims to present potential exploitations of CLDN3 and CLDN4 in the management of ovarian cancer as either biomarkers or as therapeutic targets. A comprehensive literature search was performed on PubMed to identify articles on ovarian cancer and claudin proteins. Keywords ‘claudin 3’, claudin 4’, and ‘ovarian cancer’ were used. No limits were imposed on publication time.

## Claudins as biomarkers

Claudin proteins have been identified as effective markers for the early detection, diagnosis, and prognosis of ovarian cancers. A study showed CLDN3 and CLDN4 upregulation in various subtypes of ovarian carcinoma through gene analysis [8]. Similar findings were found with immunohistochemical analysis, showing membranous and cytoplasmic elevation [9, 10]. CLDN3 and CLDN4 were also identified to have expression patterns specific to malignant ovarian tumours [10].

### Prognostic biomarkers

Several studies have explored the association between CLDN3 and CLDN4 upregulation and clinicopathological features. Such associations will aid clinicians to develop a personalised management approach by considering the extent of surgery, follow-up, and specific targeted therapies.

#### CLDN3

An immunohistochemical analysis study using 84 serous adenocarcinomas and Kaplan-Meier analysis showed that high CLDN3 expression was significantly associated with shorter patient survival [10]. However, this study found no correlation with patient age, presence of ascites, or tumour grade and stage. The study also showed that CLDN3 expression was an independent prognostic factor for predicting patient disease outcome through multivariable COX regression analysis. This contrasted with the study by Heinzelman-Schwarz et al. [18], who also used immunohistochemistry, Kaplan-Meier, and univariate Cox proportional hazards analysis on 115 primary tumour tissues and correlated to clinicopathologic features. They identified that lower CLDN3 expression was associated with poorer outcome. However, this was only a trend and not statistically significant. They also found no correlation between CLDN3 levels and relapse-free patient survival, which may have been due to the number of patients in their cohort. Also, this paper only looked at membranous CLDN3 expression and did not consider cytoplasmic staining, which may be a significant expression pattern for malignant ovarian tumours [10]. This may occur due to claudin upregulation, followed by possible dysregulations to its translocation to the membrane. Also, Davidson et al. [19] found that CLDN3 was a marker of poor overall survival when performing univariate analysis for 57 patients with disease recurrence postchemotherapy effusions. Interestingly, there was no association between CLDN3 and prechemotherapy effusions, and neither was an independent prognostic factor in recurrent disease. In a study by Kleinberg et al. [20], immunohistochemical staining of 218 effusions and 245 primary and metastatic tumours showed that CLDN3 was expressed significantly higher in the ovarian carcinoma effusions. Univariate survival analysis showed CLDN3 overexpression in effusions to correlate with shorter overall survival. The overexpression of CLDN3 was also an independent predictor of poor overall survival in postchemotherapy ovarian carcinoma effusion patients.

#### CLDN4

**Fig. 3:**
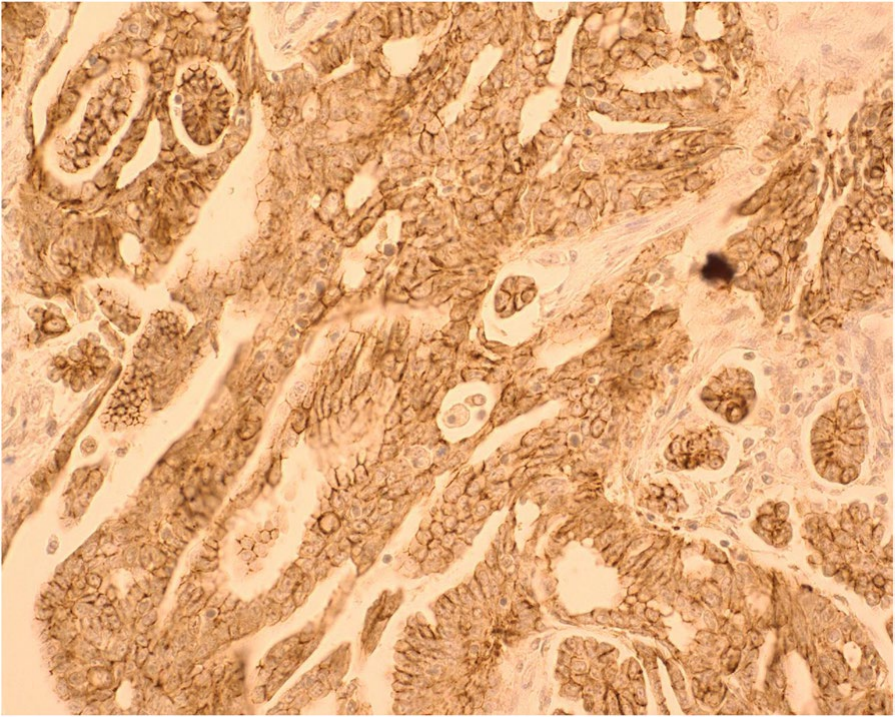
CLDN4 expression in low-grade ovarian serous carcinoma. There is membranous expression and notable cytoplasmic expression. (X200)

In the study from Choi et al. [10] for CLDN4, no significant correlation was found with the survival rate nor the patient’s clinicopathological characteristics. Interestingly, higher CLDN4 expression was found in higher grade tumours, but this was not statistically significant. This study by Kleinberg et al. [20], contrary to CLDN3, showed no significant differences between effusions and primary tumours and metastases for CLDN4. Likewise, CLDN4 did not show any correlation with disease stage or histologic grade [20]. Furthermore, CLDN4 expression in effusions was not associated with progression-free survival or overall survival. This study also did not report significant differences between primary and metastatic tissues for CLDN4 [20]. However, in the study exploring the association between CLDN4 and chemotherapy performed by Yoshida et al. [12], CLDN4 expression was associated with significantly poorer prognosis. Again, no clear correlations were observed between CLDN4 expression and other clinicopathological features. Work from our laboratory shows tumour cells may show moderate and interrupted membranous localisation and notable cytoplasmic expression of CLDN4 (Fig. 3). The figure highlights how CLDN4 is also subject to mislocalisation, with abnormal accumulation in the cytoplasm.

As many studies have highlighted the overexpression of CLDN3 and CLDN4 in ovarian carcinoma, compared to the normal ovarian surface epithelium, and some going further to highlight clinicopathological correlations, this suggests that these proteins may be useful prognostic indicators in ovarian cancer, particularly CLDN3. However, additional work in large patient cohorts is necessary to validate any correlations and verify the validity of CLDN3 as a prognostic marker.

### Diagnostic biomarkers

Considering the upregulation of CLDN3 and CLDN4 in ovarian cancer tissue, the potential application of screening for CLDN3 and CLDN4 as an alternative to CA125 to detect the cancer may be another viable route. A study found a high correlation when comparing the genetic fingerprints using oligonucleotide microarray analysis of flash-frozen OSPC with that of purified primary OSPC short-term (< 2 weeks) in vitro cultures. Therefore, the short-term in vitro culture of primary ovarian carcinomas increases the purity of RNA available, meaning that the tumour tissues’ genetic profile was preserved without significant alterations [17]. The study identified CLDN3 and CLDN4 to be the top upregulated genes, making way for this methodology to act as an early screening tool. However, despite the elevation of CLDN3 and CLND4 in ovarian carcinomas, whether the specificity and sensitivity for ovarian cancer screening in the general population are sufficient is unclear [21]. The combination of markers may be another potential route to aid the early detection process. For example, immunoperoxidase staining on 296 ovarian cancers revealed that CLDN3 was expressed in all ovarian carcinomas that lacked CA125. This suggests claudins in combination with CA125 to be potential complementary markers for ovarian cancer detection [22]. Likewise, a study that used recursive descent partition identified that CLDN3 expression levels distinguished serous, endometrioid, and clear cell from mucinous carcinomas and normal ovarian surface epithelium tissue. The tumours were then completely separated from the normal ovarian surface epithelium following further partitioning with CLDN3 and vascular endothelial growth factor (VEGF) expression levels, a signalling protein that promotes vascular growth, key for tumour progression [23]. Therefore, CLDN3 and VEGF expression together may increase the accuracy when discriminating tumour tissues from normal ovarian epithelial cells.

Since VEGF is a factor typically found in the blood, and the current gold-standard method for ovarian cancer detection is measuring CA125 levels in the blood, it begs the question as to whether claudins can also be found and measured in the serum of ovarian cancer patients. A study exploring circulating cell-free DNA by quantitative real-time PCR to assess CLDN4 as a noninvasive screening prognostic biomarker showed that the CLDN4 serum level difference observed between cases of ovarian cancer and cases with benign ovarian tumours was not statistically significant [24]. However, real-time RT-PCR analysis showed CLDN3 and CLDN4 to be upregulated in all subtypes of epithelial ovarian cancer tumour cases (serous, mucinous, clear cell, and endometrioid) when compared to the normal ovarian surface epithelia derived from the HOSE-B cell line, and ML3, a benign ovarian tumour cell line [9]. Similarly, immunohistochemical analysis showed CLDN3 and CLDN4 were also expressed in all subtypes of primary ovarian tumour cases, and no or low levels were seen in normal ovarian surface epithelium. Despite the upregulation seen in this study, the similar levels observed in the serum for CLDN4 in ovarian cancer and benign tumour cases suggest that CLDN4 may not be shed into the serum; therefore, blood screening for early detection may not be a promising method.

On the other hand, CLDN3 and CLDN4 expression can aid differential diagnosis, particularly between ovarian carcinoma and diffuse malignant peritoneal mesothelioma (DMPM). Ovarian carcinoma and DMPM can be both very similar histologically, making it difficult to diagnose accurately. Using global gene expression analysis, CLDN3, CLDN4, and CLDN6 were found to be significantly highly expressed in ovarian carcinoma compared with DMPM [25]. Therefore, molecular markers, such as CLDN3 and CLDN4, may play a crucial role in differential diagnosis alongside other markers, particularly between two closely morphologically related tumour types.

## Claudins as therapeutic targets

Around 90% of patients who respond will have disease recurrence, which usually progresses to the development of chemoresistance [3]. The 5-year survival is also at around 49% [26]. Therefore, development of alternative antitumour drugs is warranted.

### Claudin antibody therapy

Presently, antibody therapy targeting claudins has been an important area of research, with a few entering the phase I/II clinical trials, particularly those targeting CLDN18.2 [27, 28] and CLDN6 [29]. In early developmental research currently, human anti-CLDN3 IgG1 (IgGH6) antibodies have been developed. They have very high specificity to CLDN3 molecules. Confocal microscopy has shown IgGH6 to be actively internalised in the tumour cells, following native CLDN3 binding, and co-localised, probably within intracellular vesicles, with a *Clostridium perfringens* enterotoxin (CPE) peptide. Therefore, this selective uptake into tumour cells indicates a potential use for antibody-drug conjugate for therapeutic ovarian cancer applications [30]. To date, there are no clinical trials on anti-CLDN3/4 antibodies for ovarian or other cancers. However, the principle of exploiting claudin overexpression using monoclonal antibodies was utilised in a phase I/II clinical trial [NCT02054351]. Preclinical models showed IMAB027 (anti-CLDN6) to induce potent antitumour activity by antibody- and complement-dependent cytotoxicity whilst ensuring there are no off-target effects. Preliminary phase I trial data showed that IMAB027 presents a safe and well-tolerated treatment for recurrent, advanced ovarian cancer patients [31].

### Clostridium perfringens enterotoxin

**Fig. 4:**
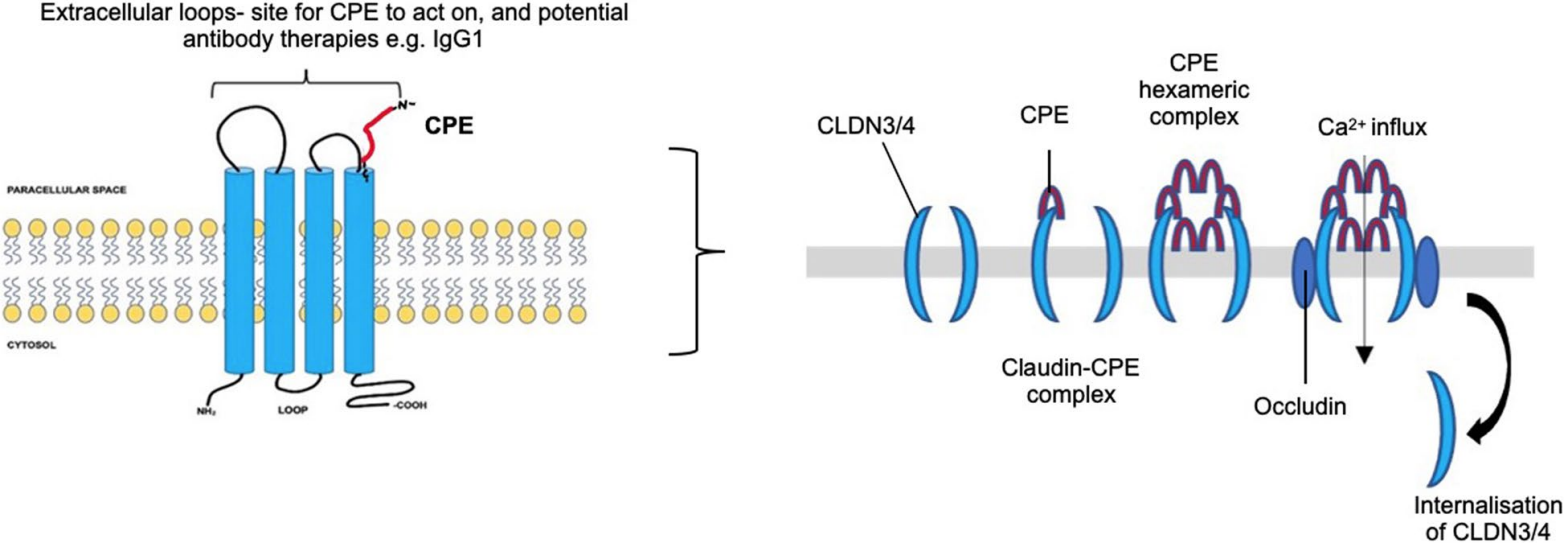
Extracellular loops represent binding sites for anti-CLDN3 antibodies, e.g., IgG1 [30] and CPE [32] (left). When CPE binds to CLDN3/4, a hexameric pore is made, compromising the cell membrane integrity. This leads to calcium ion influx and therefore cell death. The CPE-claudin complex may also incorporate occludin, thereby further breaking the TJ [32]. CPE also has been found to internalise claudins [35], further increasing the membrane’s permeability

Both CLDN3 and CLDN4 are the natural receptors for CPE. This potent toxin induces rapid cytolysis through increasing membrane permeability. They form small complexes that oligomerise and create a hexameric pore on the membranous surface, which leads to calcium influx and cell death [32]. It is also understood that occludin also participates in the breaking down of the TJ. A 30-amino acid long peptide on the C-terminus of the CPE allows it to bind to the relatively large extracellular loops on the claudins (Fig. 4) [32]. Therefore, considering CLDN3 and CLDN4 abundance in ovarian cancer, several studies have highlighted CPE as a potential antitumour drug. Proof-of-principle demonstration for CPE use as a chemotherapeutic agent has been successful in in vitro studies [33]. Through in situ mRNA hybridisation, CLDN3 expression was found in vivo in human prostate carcinoma epithelium. The level of cytotoxicity correlated with CLDN3 overexpression in a primary culture of metastatic prostatic adenocarcinoma treated with CPE [34]. This toxicity suggests CPE to likely be cytotoxic in vivo and therefore act as an alternative to chemotherapy.

Further cell culture work has found CLDN4 also to be the main target for CPE. In another study using CLDN4 overexpressing human pancreatic cancer cell lines, an acute dose-dependent cytotoxic effect was discovered when treated with CPE [36]. This was interestingly restricted to CLDN4 expressing cells, and the strength of CLDN4 expression determined the magnitude of the effects. The toxicity was determined in vitro by trypan blue exclusion and the ^86^Rb-release assay. This study also went further and assessed CPE’s activity in vivo, where CPE intramural injection on nude mice with PANC-1 cell line xenografts demonstrated extensive tumour cell area necrosis and tumour growth reduction.

The effects of CPE are further highlighted in another study utilising chemosensitive and chemoresistant tumour samples. Fresh human ovarian cancer cell lines and established cancer cell lines were used to evaluate CLDN3 and CLDN4 expression by real-time qRT-PCR. Established ovarian cancer cell line OVA-1 and a fresh OSPC, found to be chemoresistant, were used to establish ovarian xenografts in severe combined immunodeficient (SCID) mice [13]. The study demonstrated that multiple intraperitoneal CPE administrations at sublethal doses lead to significant tumour growth inhibition in all SCID mouse xenografts, thereby circumventing the initially chemoresistant properties of the xenograft tissue. Furthermore, tumour progression inhibition and survival extension were also induced by CPE. This phenomenon, however, occurred regardless of chemoresistance or chemosensitivity. Moreover, most of the mice that harboured OVA-1 xenografts and treated with repeated intraperitoneal CPE injections remained alive and free of any detectable tumour for over 120 days. Therefore, the findings of this study indicate that CPE-based therapy is an effective and beneficial treatment to ovarian cancer patients refractory to conventional treatment modalities. An exciting phenomenon seen in this study [13] is that CLDN3 and CLDN4 were also overexpressed at significantly higher levels in chemoresistant/recurrent tumours in contrast to chemosensitive tumours, concordant with previous studies [12, 15].

### Chemotherapy and CPE

While more studies are required to clear the controversy between the expression patterns of CLDN3, CLDN4, and chemotherapy efficacy, the use of CPE to sensitise chemoresistant cells was explored. Gao et al. [35] showed that CPE infusion in a three-dimensional epithelial ovarian cancer culture model, developed using SKOV-3 and RMUG-L cell lines, downregulated CLDN4 and translocated it to the cytoplasm (Fig. 4). This was also evident in Madin-Darby canine kidney and Caco-2 colorectal carcinoma cells, where CLDN4 was specifically disintegrated and relocated after CPE administration, thereby diminishing TJ function. Analysis through qRT-PCR showed that the cell line OVCA-429 expressed the highest levels of CLDN4 mRNA, followed by SKOV-3, RMUG-L, and TOV112D expressing the lowest. Tumour growth inhibition and sensitisation, as measured by MTT assay, in response to Taxol and carboplatin, combined with CPE, was greatest in the OVCA-429 cell line [35]. This may be in response to the reduced barrier function due to reduced CLDN4 expression after CPE injection and therefore increased drug penetration and accumulation in the tumour core. This theory is further supported by the fact that SKOV-3 cell lines responded less, and TOV112D was completely resistant, as measured by cell growth and viability assays. Interestingly, for SKOV-3, there was no enhanced antitumour effect with carboplatin, despite a significant antitumour response with Taxol. This may be due to reduced CLDN4 levels and carboplatin being less potent than Taxol. Furthermore, the study using mice bearing SKOV-3 xenografts also showed that repeated intraperitoneal CPE injections could sensitise epithelial ovarian cancer cells to low-dose Taxol, thereby suppressing large tumour burdens in vivo [35]. This is concordant to Santin et al. [13] findings where multiple intraperitoneal administrations lead to a decreased tumour burden. Moreover, oligonucleotide microarray analysis between CPE-treated and control SKOV-3 cells showed that CPE treatment induced upregulation of genes such as NADH dehydrogenase (ubiquinone) 1 beta subcomplex and glutaminyl-peptide cyclotransferase-like, both important for intracellular protein degradation, receptor signalling regulation, proliferation, angiogenesis, and apoptosis. They have also been identified to attenuate molecules such as phosphoglucomutase 1, important for cellular metabolism. Therefore, it is understood that stimulation of the ubiquitin-proteasome pathways by CPE may contribute to the increased sensitivity of the tumours to chemotherapy.

In an in vitro study using human ovarian cancer cell lines, small-hairpin RNA and CLDN4 mimic peptide were used to silence CLDN4 gene expression and inhibit CLDN4 activity, respectively [15]. This interestingly improved the apoptotic response to paclitaxel in human-derived OVCAR-3 and PEO-4 ovarian tumour cells. OVCAR-3 cells with reduced CLDN4 proliferated more slowly with enhanced mitotic arrests when compared to their CLDN4 overexpressing cells. Furthermore, they identified that in OVCAR-3 cells, CLDN4 seemed to interact with the tubulin, thereby having a profound effect on the microtubular network polymerisation and structure. Consequently, reducing CLDN4 activity influenced the cells’ increased response to paclitaxel. Since the TJ is maintained throughout the cell cycle and cell division, CPE can deliver anticancer drugs at all times of the cell cycle. This is clinically important, as many chemotherapeutic agents require the cell to be at a specific cell cycle phase, such as paclitaxel’s action during the M phase. Therefore, the increased sensitivity to chemotherapy after CLDN3 and CLDN4 dysregulation highlights their potential as therapeutic targets.

### CPE and imaging

In addition to its potential as an anticancer therapy, CPE has also been shown to be a promising tool for fluorescence imaging systems, important for patient management when receiving neoadjuvant chemotherapy [37]. This has been shown to play a vital role during interval debulking. Fluorescein isothiocyanate (FITC) can conjugate with CPE (FITC-c-CPE); this is capable of binding and internalising into many CLDN3 and CLDN4 expressing ovarian carcinoma cells both in vitro and in vivo. The conjugate also binds rapidly to tumours. This method is highly sensitive to the visualisation of peritoneal micrometastatic tumour implants and identifying ovarian tumour spheroids in malignant ascites in vivo in real time that can otherwise be missed by conventional visual observation. Furthermore, such optical methods have allowed for the conjugation of gold nanoparticles to CPE, which subsequently binds specifically to claudins. In an in vitro study, through utilising gold nanoparticle-mediated laser perforation techniques, ablation of cells derived from human and canine tumour cell lines was possible, eliminating more than 75% of claudin overexpressing cells and not majorly interfering with claudin non-expressing cells [38]. Therefore, both studies highlight the role of CPE in developing a practical optical approach in primary debulking surgery and identification of residual disease after neoadjuvant chemotherapy treatment.

### Limitations of CPE therapy

Therapeutic approaches under development that utilise CLDN3 and CLDN4 seem primarily focused on CPE; its direct cytotoxic effect, ability to sensitise chemoresistant cancers, and screening/image-directed therapy capabilities justify the necessity to investigate this molecule further. Two studies administered CPE multiple times [13, 35], which may encourage the formation of neutralising antibodies, thereby reducing efficacy of CPE [39]. However, considering the immune dysregulation within the peritoneal cavity of advanced stage ovarian cancer patients, this may be overcome [39]. Also, the anti-enterotoxin antibodies are not made and released in time to prevent the consequences of CPE ingestion [39]. Therefore, these findings support the notion that the immune system may not impair the activity of CPE [39]. However, more clinical studies are warranted to accurately determine the immune response and, therefore, the efficacy against CPE in human patients.

Generally, intraperitoneal administration is preferred over intravenous administration due to significantly fewer adverse outcomes. However, this route requires the even distribution of the toxin throughout the abdominal cavity to reach the tumour tissue. Since many elderly ovarian cancer patients would have undergone surgery and subsequent adhesions, these may prevent the homogenous distribution of CPE therapy. This will reduce its localised efficacy [13]. Moreover, CPE will likely favour intraperitoneal ovarian tumour plaques through passive diffusion, as the distance is only a few millimetres. This means that local CPE administration in patients with a significant tumour burden will have reduced efficacy, due to its inability to deeply penetrate large tumour masses. This suggests that local CPE administration would mainly benefit patients with either microscopic residual disease or small-volume macroscopic cancers that are resistant to standard chemotherapy [13]. This, therefore, promotes the idea of downregulating CLDN3 and CLDN4 to sensitise the cells to chemotherapeutic agents using CPE. This is further supported by using CPE at high doses for a short period of time, leading to better efficacy and fewer adverse events. Furthermore, as this therapy is not reliant upon the immune system, it is beneficial for elderly ovarian cancer patients undergoing immunosuppressive chemotherapy [13]. Consequently, whilst CPE usage seems promising as an ovarian cancer therapy, it still requires phase I and phase II clinical trials, necessary to determine the feasibility of this therapeutic approach.

## Conclusions

CLDN3 and CLDN4 have been shown to be overexpressed in ovarian cancers. The overexpression of these claudins, particularly CLDN3, has been shown by several studies to be associated with poorer survival outcomes. Both CLDN3 and CLDN4 are potential therapeutic targets, with CLDN3 showing potential in antibody therapy and CLDN4 with CPE therapy. With the promising results of in vitro and in vivo studies, further research in phase I and II clinical trials is required to validate these findings in a clinical setting with potential to add to the strategies of ovarian cancer management.

## Data Availability

Not applicable.

## Abbreviations

CEA: Carcinoembryonic antigen A
CA-125: Cancer antigen 125
CA 19-9: Carbohydrate antigen 19-9
HE4: Human epididymis 4
CLDN3: Claudin-3
CLDN4: Claudin-4
(q)RT-PCR: (Quantitative) reverse transcription-polymerase chain reaction
VEGF: Vascular endothelial growth factor
HOSE: Human ovarian surface epithelium
DMPM: Diffuse malignant peritoneal mesothelioma
CPE: *Clostridium perfringens* enterotoxin
OSPC: Ovarian serous papillary carcinoma
MTT: 3-(4,5-Dimethylthiazol-2-yl)-2,5-diphenyltetrazolium bromide
FITC: Fluorescein isothiocyanate
